# Initial diagnosis patterns of coexisting mental health and neurodevelopmental conditions in autistic children and youth: Evidence from a nationally representative sample in Canada

**DOI:** 10.1111/jcpp.70039

**Published:** 2025-09-01

**Authors:** Yun‐Ju Chen, Meng‐Chuan Lai, Stelios Georgiades, Eric Duku, Jordan Edwards, Emma Nolan, Peter Szatmari, Ryan Miller, Katherine Cost, Katholiki Georgiades

**Affiliations:** ^1^ Offord Centre for Child Studies McMaster University Hamilton Ontario Canada; ^2^ Department of Occupational Therapy, Graduate Institute of Behavioral Sciences College of Medicine, Chang Gung University Taoyuan Taiwan; ^3^ Department of Psychiatry Chang Gung Memorial Hospital at Linkou Taoyuan Taiwan; ^4^ Campbell Family Mental Health Research Institute, Centre for Addiction and Mental Health Toronto Ontario Canada; ^5^ Department of Psychiatry Temerty Faculty of Medicine, University of Toronto Toronto Ontario Canada; ^6^ Department of Psychology, Faculty of Arts and Science University of Toronto Toronto Ontario Canada; ^7^ Department of Psychiatry The Hospital for Sick Children Toronto Ontario Canada; ^8^ Department of Psychiatry, Autism Research Centre University of Cambridge Cambridge UK; ^9^ Department of Psychiatry National Taiwan University Hospital and College of Medicine Taipei Taiwan; ^10^ Hamilton Health Sciences Hamilton Ontario Canada; ^11^ School of Psychology Queen's University Belfast Belfast Northern Ireland UK; ^12^ Department of Psychology University of Waterloo Waterloo Ontario Canada

**Keywords:** Autism, age of diagnosis, neurodevelopmental conditions, mental health, sex differences

## Abstract

**Background:**

Elevated prevalence of coexisting health conditions has been observed in autistic people, yet how the timing of their initial diagnoses varies by sex and age of autism diagnosis remains understudied. Using a person‐centered approach, we examined the patterns of initial diagnosis for mental health and neurodevelopmental conditions among autistic children and youth identified from the general population.

**Methods:**

The sample was drawn from the 2019 Canadian Health Survey on Children and Youth (CHSCY) cohort (*N* = 47,781), consisting of 776 5–17‐year‐olds (82% assigned‐male‐at‐birth) with a caregiver‐reported diagnosis of autism. Multigroup latent class analysis was used to identify subgroups based on ages of initial diagnoses of autism, anxiety, mood, learning, and attention‐deficit/hyperactivity disorders stratified by sex assigned at birth. Functional difficulties and multimorbidity status, including the number and types of coexisting conditions, were compared across the subgroups.

**Results:**

Four latent subgroups were identified for each sex group, primarily differentiated by the age of autism diagnosis. The most prevalent class (46%) was characterized by an initial autism diagnosis at ages 3–5 years. The remaining subgroups, with autism diagnosed primarily before age 3, at 6–8, and at 9–17 years, each comprised ~20% of the sample. Subgroups with autism diagnosed after age 6 tended to have more coexisting conditions, with females showing heightened probabilities of mental health diagnoses across age windows from birth to age 17 years. The temporal order of coexisting diagnoses relative to autism diagnosis varied across subgroups, with sex differences more evident for anxiety and attention‐deficit/hyperactivity disorders.

**Conclusions:**

There were nuanced variations in the timing of initial diagnoses of coexisting conditions based on the age of autism diagnosis. The sex‐varying patterns highlight the importance of continuous monitoring and evaluation of the neurodevelopmental and mental health needs of autistic children and youth, with supports tailored to sex and the timing of autism diagnosis.

## Introduction

A major challenge faced by autistic people is the heightened likelihood of having additional health conditions, which significantly impact one's daily functioning, quality of life, and overall well‐being (Lai, 2023; Lord et al., 2022; Menezes & Mazurek, 2021). A recent national survey in Canada reveals that approximately 70% of autistic children and youth have at least one additional diagnosis (Public Health Agency of Canada, 2022), aligning with previous estimates from the United States and United Kingdom (Levy et al., 2010; Simonoff et al., 2008). Other neurodevelopmental conditions, such as attention‐deficit/hyperactivity disorder (ADHD) and learning disorders/disabilities (LD), have prevalence estimates exceeding 30%, while mental health conditions, such as anxiety disorders, affect over 20% of the autistic population (Lai et al., 2019; Micai et al., 2023; Public Health Agency of Canada, 2022; Simonoff et al., 2008). Such evidence highlights the significant health needs of autistic people and the importance of prioritizing these needs when assessing long‐term outcomes and planning support and services (Lai, 2023; Lord et al., 2022; Waizbard‐Bartov, Fein, Lord, & Amaral, 2023). Given the variability in the prevalence estimates of coexisting conditions due to different sampling strategies and ascertainment methods (Lai et al., 2019; Smith et al., 2024), it is crucial to obtain evidence from the general population, which is currently limited.

Lifetime prevalence estimates of coexisting health conditions in autism provide essential evidence of the wide‐ranging challenges and support needs of autistic people and their families. However, it does not offer insights into the emergence and developmental course of these health conditions, which hold significant implications for prevention, screening, early detection, and early intervention. Further, the lifetime prevalence is often estimated separately for each condition, which is limited in capturing the simultaneous or successive occurrence of multiple conditions. For instance, the co‐occurrence of autism and ADHD is commonly documented and can mutually overshadow symptoms, complicating the diagnostic process (Kentrou, de Veld, Mataw, & Begeer, 2019; Lai, Lin, & Ameis, 2022; Miodovnik, Harstad, Sideridis, & Huntington, 2015). While previous research on the patterns of coexisting health conditions in autism has revealed distinct subgroups based on the presence and severity of coexisting physical and psychiatric conditions (Doshi‐Velez, Ge, & Kohane, 2014; Miot et al., 2023; Piergies, Hirota, Monden, & Zheng, 2022), the *temporal* aspect of co‐occurrence has rarely been studied. Sex and gender also matter in understanding the patterns of coexisting health conditions in autistic people (Bölte et al., 2023). In both general and autistic populations, research commonly shows that diagnoses of neurodevelopmental and behavioral conditions, such as ADHD and conduct disorders, are more prevalent in males, while females are more likely to be diagnosed with emotion‐related conditions, such as anxiety and mood disorders (Angell et al., 2021; Höglund, Hakelind, & Nordin, 2020; Hull, Mandy, & Petrides, 2017; Kessler & Wang, 2008; Rødgaard, Jensen, Miskowiak, & Mottron, 2021). However, sex differences in mental health can be developmentally variable due to the interplay of biological and socio‐ecological factors, particularly after puberty (Bölte et al., 2023; Lai, 2023). Diagnostic overshadowing and camouflaging/masking of autistic traits, which are reported in some autistic females and may be linked with gender socialization experiences, can further lead to delayed identification and diagnosis of autism and contribute to mental health difficulties (Lai et al., 2022, 2023; Wodka et al., 2022). Recent evidence has shown that females, on average, tend to be diagnosed with autism at a later age than males (Milner et al., 2023; Smith et al., 2024), but this sex difference may not hold when accounting for coexisting conditions or intellectual functioning (Gu, Dawson, & Engelhard, 2023; Harrop et al., 2021). These findings highlight the necessity of considering sex differences to better understand the variability in the timing of diagnoses of autism and coexisting conditions.

Using latent class analysis, a person‐centered approach to identifying homogeneous subgroups or patterns (Collins & Lanza, 2009), the current study aimed to identify patterns based on the ages of initial diagnoses of autism and coexisting neurodevelopmental and mental health conditions. Age‐specific presence or absence of a caregiver‐reported first diagnosis for each condition was used as indicators for subgrouping. This approach helps identify critical age windows associated with an increased likelihood of diagnosis for specific conditions, while accommodating individual variability in these timelines. Using general population‐based data from 776 children and youth aged 5–17 years with a caregiver‐reported autism diagnosis, drawn from a nationally representative sample in Canada, we addressed the following aims:

1. To identify subgroups based on the age of initial diagnoses of autism and four commonly coexisting conditions, including anxiety disorders, mood disorders, LD, and ADHD in autistic males and females.
2. To examine the extent to which these subgroups differed in terms of current functional difficulties and multimorbidity, which includes the number and types of coexisting conditions.

Considering the significant associations observed between coexisting conditions, sex, and age of autism diagnosis in previous research (Jadav & Bal, 2022; Smith et al., 2024), we anticipated that the age at the initial autism diagnosis would be a strong indicator for subgrouping, with differing patterns of coexisting diagnoses by the timing of autism diagnosis and sex. We also expected that the identified subgroups would vary by their current functioning, aligning with previous research documenting the significant impacts of coexisting conditions on the daily functioning of autistic children and youth (Lai, 2023; Lord et al., 2022; Menezes & Mazurek, 2021).

## Method

### Participants and procedure

The current sample was drawn from the 2019 Canadian Health Survey on Children and Youth (CHSCY), a cross‐sectional national survey of 47,781 children and youth aged 1–17 years and their caregivers living in private dwellings across 10 provinces and three territories in Canada (Statistics Canada, 2019). The 2018 Canadian Child Benefit file was used as the sampling frame, covering 98% of children and youth living in the provinces and 96% in the territories. Age stratification and geographical sub‐stratification were employed to construct a representative sample of the Canadian children and adolescent population. The survey was administered through electronic questionnaires and/or phone interview follow‐ups between 11 February 2019 and 2 August 2019, with an overall response rate of 52%. Sampling weights were calculated to account for out‐of‐scope units, nonresponse, extreme weight trimming, and calibration to known population totals. The current study focused on 5–17‐year‐olds at the time of sampling, as several variables of interest regarding health condition diagnoses were only available for this age range. The analytic sample comprised 2% of the 2019 CHSCY sample aged 5–17 years (*N*_unweighted_ = 776, *N*_weighted_ = 112,707) with a caregiver‐reported autism diagnosis.

### Measures

#### Latent class indicators

The primary outcomes of interest were derived from caregiver‐reported questions about the age (in years) at which autism and the following four types of neurodevelopmental and mental health conditions were *initially* diagnosed by a health professional.

Mental health conditions:

- Anxiety disorders (including phobia, obsessive‐compulsive disorder, or panic disorder)
- Mood disorders (including depression, bipolar disorder, mania, or dysthymia)

Neurodevelopmental conditions:

- Learning disorders or learning disabilities
- Attention‐deficit disorder or ADHD

The age of diagnosis for autism and each of these four conditions was recoded into binary indicators representing the presence or absence of an initial diagnosis within each of the six following age windows: birth to 2, 3–5, 6–8, 9–11, 12–14, and 15–17 years. If the target age window exceeded the child's age at the time of the survey, the indicators were coded as missing to reflect that the child had *not yet* received a diagnosis. If a child was reported to be diagnosed at a specific age, the corresponding age window was coded as 1, and subsequent age windows were coded as 0, since the *initial* diagnosis can only occur once in a lifetime. The recoding resulted in a total of 30 indicators (i.e., five conditions × six age windows), serving as the primary indicators for latent class analysis.

#### Associated outcomes

##### Multimorbidity status

Multimorbidity status was operationalized as the total number of caregiver‐reported diagnoses out of the four conditions (count variable; range = 0–4) and the following three binary variables that indicate the presence or absence of (1) coexisting mental health conditions (i.e., anxiety and/or mood disorders) only, (2) coexisting neurodevelopmental conditions (i.e., LD and/or ADHD other than autism) only, and (3) coexisting neurodevelopmental and mental health conditions (coexisting MH‐NDD). These conditions, along with autism, are all recognized as chronic in the CHSCY survey (Statistics Canada, 2019), defined as lasting or expected to last 6 months or more and diagnosed by a health professional. Accordingly, they were treated in this analysis as chronic contributors to *lifetime* multimorbidity status, irrespective of current symptom severity or diagnostic status.

##### Functional difficulties

The current analysis used a modified 5–17‐year‐old version of the Washington Group/UNICEF Child Functioning Module (WG/UNICEF CFM; Washington Group on Disability Statistics, 2017) to assess functional difficulties at the time of the survey. This modified version included 10 caregiver‐reported items, excluding those assessing the functional domains of vision, hearing, and mobility, as the item structure and response options for these domains differ from all others. Functional difficulties in cognitive, behavioral, and interpersonal domains were rated on a 4‐point scale and recoded per WG/UNICEF CFM guidelines (Washington Group on Disability Statistics, 2017): 0 = no or some difficulty; 1 = a lot of difficulty or cannot do at all. Items measuring difficulties related to mood and anxiety were assessed on a 5‐point response scale (recoded as 0 = never/a few times a year/monthly/weekly; 1 = daily). Confirmatory factor analysis (CFA) of 10 binary‐coded items in the current autistic sample revealed a three‐factor structure: (1) Cognitive Difficulties (5 items), which included items assessing self‐care, communication, learning, remembering, and concentrating; (2) behavioral‐interpersonal difficulties (3 items), which included items assessing accepting change, controlling behavior, and making friends; and (3) Emotional Difficulties (2 items), which included items assessing anxiety and depression. This 3‐factor model demonstrated excellent model fit for the current autistic sample, supporting its structural validity: Comparative Fit Indices (CFI) = 0.99, Tucker‐Lewis Index (TLI) = 0.99, and Root Mean Square Error of Approximation (RMSEA) = 0.02. The item factor loadings are provided in Table S1. Children and youth who were reported to have a functional difficulty in at least one item within each latent factor, as well as across all three factors, were classified as having a functional difficulty for that factor and overall.

### Statistical analysis

To identify patterns of initial diagnoses of autism and the four commonly coexisting health conditions across the six age windows (i.e., birth to 2, 3–5, 6–8, 9–11, 12–14, and 15–17 years), multigroup latent class analysis was conducted using the recoded binary indicators of initial diagnosis for autism and the four health conditions as indicators. Sex assigned at birth was included as the grouping variable. Before fitting multigroup models, single‐group latent class models with varying numbers of classes were fitted to explore the general pattern of clustering. A series of multigroup latent class models with two to five classes were then fitted, including: (1) unconstrained models allowing class sizes and conditional response probabilities to differ across sex groups and (2) partially constrained models that fixed the probabilities of autism diagnosis constant across sex groups to facilitate interpretation (i.e., how the initial diagnosis patterns of other conditions vary by sex, given that the likelihood of the initial autism diagnosis is held constant). The optimal number of classes was determined using Bayesian information criterion (BIC) and sample size‐adjusted BIC (SABIC), where lower values indicate better model fit and parsimony, along with entropy, where higher values close to 1 indicate greater classification certainty (Geiser, Lehmann, & Eid, 2006).

Upon identifying the optimal class solution, Wald chi‐squared tests were used to assess overall differences across the latent classes in the associated outcomes, including multimorbidity status and current functional difficulties. Post‐hoc regression models with analytic sampling weights were then implemented using the most prevalent subgroup (i.e., male Class 2) as the reference subgroup for groupwise comparisons, while controlling for age at sampling. Further, the age at diagnosis for each of the four conditions relative to the autism diagnosis was compared across the latent classes to clarify the temporal order of the initial diagnoses.

Latent class analyses were performed using Mplus 8.7 (Muthén & Muthén, 2017), with missing data handled with full information maximum likelihood estimation. Subsequent regression analyses were conducted with Stata 17 (StataCorp, 2021), applying listwise deletion to missing outcome variables (rates of missingness < 1%). Sampling weights were applied throughout the analyses to obtain weighted estimates for population inference.

## Results

Table 1 presents the weighted descriptive statistics for sample demographics, diagnostic status, and functional difficulties with sex stratification. Among the autistic children and youth in the CHSCY sample, 68.5% had at least one of the four coexisting conditions. The prevalence estimates across the sample age range of 5–17 years were 21.8% for anxiety disorders, 6.3% for mood disorders, 48.7% for LD, and 40.6% for ADHD. The mean ages at diagnoses (with standard deviations) were 5.6 (3.4) years for autism, 6.9 (3.3) years for anxiety disorders, 9.3 (4.3) years for mood disorders, 5.3 (3.0) years for LD, and 6.1 (3.0) years for ADHD. There was a significantly higher prevalence of anxiety disorders in autistic females compared to autistic males (35.0% vs. 18.8%; *χ* ^2^ = 18.6, *p <* .001). No significant sex differences were observed in the prevalence and ages at diagnosis for autism and the other coexisting conditions.

**Table 1:** Weighted sample characteristics overall and stratified by sex assigned at birth, of children and youth with a reported autism diagnosis in CHSCY 2019

	All	Male	Female
Demographics			
*N* (%)	776 (100)	627 (81.55)	149 (18.45)
Age in years, mean (*SD*)	10.68 (3.83)	10.72 (3.85)	10.48 (3.79)
Racial/ethnic minority status, %			
Racialized identity^a^	22.57	23.85	16.95
Indigenous identity	7.34	7.51	6.58
Born in Canada, %	91.54	90.27	97.12
Primary caregiver's highest education, %			
High school degree or below	26.15	25.57	28.69
Some post‐secondary education	40.49	40.73	39.47
Bachelor's degree or above	33.36	33.70	31.84
Household income in CAD, mean (*SD*)	91,548.29 (75,175.16)	94,108.68 (77,429.73)	80,232.91 (63,040.68)
Latent class indicators and associated outcomes			
Presence of coexisting diagnosis, %			
Any diagnosis^b^	68.46	67.52	72.63
Anxiety disorders	21.79	18.77	35.03
Mood disorders	6.29	6.06	7.33
Learning disorders	48.69	48.36	50.15
ADHD	40.56	41.15	37.96
Age at diagnosis, mean (*SD*)			
Autism	5.56 (3.42)	5.59 (3.37)	5.42 (3.61)
Anxiety disorders	6.87 (3.33)	6.88 (3.43)	6.83 (3.10)
Mood disorders	9.32 (4.31)	9.18 (4.29)	9.78 (4.38)
Learning disorders	5.30 (3.02)	5.40 (3.02)	4.86 (2.97)
ADHD	6.12 (3.01)	6.07 (2.82)	6.35 (3.79)
Presence of functional difficulties, %			
Cognitive	44.83	42.69	54.38
Behavioral‐Interpersonal	65.46	64.33	70.43
Emotional	30.33	29.46	34.15
Any	75.29	74.26	79.81

ADHD, attention‐deficit/hyperactivity disorder; *SD*, standard deviation.

^a^ The visible minority population, as defined by Statistics Canada, primarily includes South Asian, Chinese, Black, Filipino, Arab, Latin American, Southeast Asian, West Asian, Korean, and Japanese.

^b^ Presence of any diagnosis out of the four coexisting conditions.

### Latent classes of initial diagnosis patterns

As shown in Table 2, the reductions of BIC and SABIC decelerated beyond the 4‐class solutions across single‐group and multigroup models. The partially constrained models generally fit better than the unconstrained models, indicating a preference for parsimony in the latent class structure across sex. Based on the model fit statistics and clinical interpretations of the latent class models, a partially constrained four‐class multigroup model was selected as the optimal solution for subsequent analyses.

**Table 2:** Model fit statistics of latent class models with different class numbers and constraints

Number of classes	BIC	SABIC	Entropy	Class proportions (%)	Elbow plot for the information criteria
Unconstrained single‐group					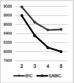
2	8,990.47	8,796.77	1	44/56	
3	8,648.56	8,356.42	1	22/34/44	
4	8,481.20	8,090.62	0.997	15/19/22/44	
5	8,490.12	8,001.10	0.992	8/11/15/21/45	
Unconstrained multigroup					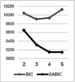
2	10,044.24	9,653.66	1	40/60	
3	9,906.35	9,318.89	0.982	26/33/41	
4	9,934.83	9,150.49	0.989	18/19/22/41	
5	10,126.81	9,145.59	0.984	13/14/16/19/38	
Partially constrained multigroup (fixed probabilities of autism diagnosis across sex)					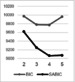
2	9,974.66	9,622.18	1	44/56	
3	9,783.42	9,253.12	0.993	21/35/44	
4	**9,773.62**	**9,065.49**	**0.992**	**16/19/21/44**	
5	9,961.98	9,076.02	0.971	11/12/15/19/43	

The selected model is highlighted in bold. BIC, Bayesian information criterion; SABIC, sample size‐adjusted Bayesian information criterion.

**Figure 1:**
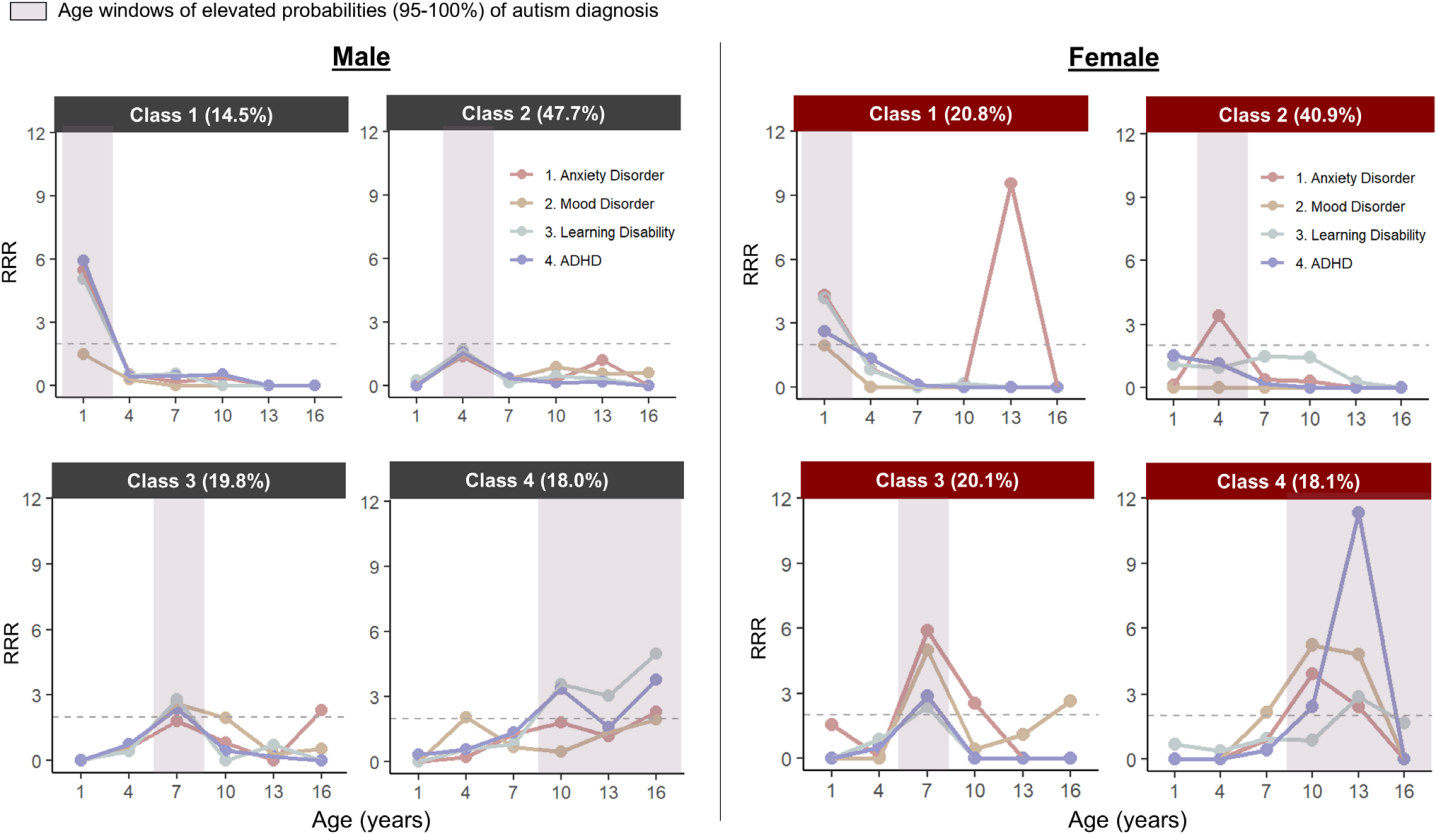
Relative rate ratios (RRRs) of initial diagnoses of coexisting conditions by latent class. The RRRs were calculated by dividing the class‐specific probability estimates (as reported in Table 3) by the overall probability estimates across the entire autistic sample (*N* = 776). The prevalence of each class within each sex is shown in parentheses. The dashed horizontal line indicates the cutoff of RRR = 2 for clinically significant elevated rates of initial diagnosis, meaning the initial diagnosis rate for that subgroup is more than twice as high as the rate for the overall autistic population at a specific age window

The estimated weighted probabilities of initial diagnoses across the six age windows for each condition from the 4‐class model are reported in Table 3. To gain deeper insights into the clinical relevance of the probability estimates, Figure 1 plots the *y*‐axis as the relative rate ratios (RRRs), calculated by dividing the class‐specific probabilities by the overall probabilities across the entire autistic sample. An RRR value of 2 was set as the cutoff for indicating clinical significance (Andrade, 2015). The latent classes were primarily differentiated by the timing of autism diagnosis, with class prevalence ranging from 16% to 46%:

- Class 1 (16%; *N*_unweighted_ = 122) is characterized by a 95% probability of an initial autism diagnosis between birth and age 2 years. Elevated probabilities of initial diagnoses for LD (29.3%–35.6%; RRRs = 4.2–5.1), ADHD (10.7%–24.2%; RRRs = 2.6–5.9), and anxiety disorders (3.9%–4.9%; RRRs = 4.3–5.4) were observed in both male and female groups from birth to age 2 years. Additionally, an elevated probability of initial anxiety disorder diagnosis was noted in the female group at ages 12–14 years (38.2%; RRR = 9.6). A larger proportion of females (20.8% of all autistic females) were classified into this class than males (14.5% of all autistic males).
- Class 2 (46%; *N*_unweighted_ = 360) is the most prevalent class, characterized by a 100% probability of an initial autism diagnosis between ages 3 and 5 years. The RRR values for initial diagnoses of coexisting conditions were generally below 2, indicating consistency with the overall age‐specific estimates across the entire autistic sample, except for an elevated probability of initial diagnoses of anxiety disorders in the female group at ages 3–5 years (27.9%; RRR = 3.4).
- Class 3 (20%; *N*_unweighted_ = 154) is characterized by a 100% probability of an initial autism diagnosis between ages 6 and 8 years. For the male group, elevated probabilities of initial diagnoses for LD (37.9%; RRR = 2.8), ADHD (45.9%; RRR = 2.4), and mood disorders (3.1%; RRR = 2.6) were observed at ages 6–8 years, while an elevated probability of anxiety disorder diagnoses was noted between ages 15 and 17 years (2.3%; RRR = 2.5). For the female group, elevated probabilities of initial diagnoses for LD (55.3%; RRR = 2.4), ADHD (32.3%; RRR = 2.8), anxiety disorders (36.5%; RRR = 5.9), and mood disorders (6.0%; RRR = 5.0) were observed at ages 6–8 years. Additionally, there were elevated probabilities of anxiety disorders observed at ages 9–11 years (14.4%; RRR = 2.5) and mood disorders at ages 15–17 years (7.9%; RRR = 2.6).
- Class 4 (18%; *N*_unweighted_ = 140) is characterized by a 95% probability of initial autism diagnosis at age 9 and beyond. For the male group, elevated probabilities of initial diagnoses for LD (3.0%–21.1%; RRRs = 3.0–5.0) and ADHD (3.8%–19.2%; RRRs = 1.6–3.8) were observed at ages 9–17 years. For the female group, elevated probabilities of anxiety (9.7%–22.5%; RRRs = 2.4–3.9) and mood disorders (8.9%–15.9%; RRRs = 4.8–5.2) were observed at ages 9–14 years, with a particularly high probability of ADHD diagnosis at ages 12–14 years (35.1%; RRR = 11.3).

**Table 3:** The estimated weighted probabilities (%) of initial diagnoses by age window for each condition from the 4‐class model

	Class 1				Class 2				Class 3				Class 4			
	Male		Female		Male		Female		Male		Female		Male		Female	
Class prevalence (%)	14.5		20.8		47.7		40.9		19.8		20.1		18.0		18.1	
*N*_unweighted_	91		31		299		61		124		30		113		27	
Age in years	Prob.	95% CI	Prob.	95% CI	Prob.	95% CI	Prob.	95% CI	Prob.	95% CI	Prob.	95% CI	Prob.	95% CI	Prob.	95% CI
Autism																
Birth–2	95.1	91.0, 99.2	95.1	91.0, 99.2	0	0, 0	0	0, 0	0	0, 0	0	0, 0	0.1	0, 0.2	0.1	0, 0.2
3–5	0.3	0, 0.6	0.3	0, 0.6	100	100, 100	100	100, 100	0	0, 0	0	0, 0	0.1	0, 0.2	0.1	0, 0.2
6–8	0	0, 0	0	0, 0	0	0, 0	0	0, 0	0	0, 0	0	0, 0	2.9	1.2, 4.6	2.9	1.2, 4.6
9–11	0	0, 0	0	0, 0	0	0, 0	0	0, 0	100	100, 100	100	100, 100	58.1	51.8, 64.4	58.1	51.8, 64.4
12–14	2.6	0, 5.9	2.6	0, 5.9	0	0, 0	0	0, 0	0	0, 0	0	0, 0	25.8	20.0, 31.6	25.8	20.0, 31.6
15–17	0	0, 0	0	0, 0	0	0, 0	0	0, 0	0	0, 0	0	0, 0	11.4	7.0, 15.8	11.4	7.0, 15.8
Avg. age at diagnosis in years (*SE*)	1.97 (.01)		1.60 (.01)		3.85 (.00)		3.79 (.01)		6.87 (.01)		6.81 (.01)		11.13 (.02)		11.99 (.04)	
Anxiety disorders																
Birth–2	4.9	2.1, 7.7	3.9	0, 7.8	0.1	0, 0.2	0.1	0, 0.2	0	0, 0	1.4	0, 2.8	0	0, 0	0	0, 0
3–5	4.3	0.5, 8.1	7.1	0.3, 13.9	11.4	8.6, 14.2	27.9	17.7, 38.1	4.8	2.9, 6.7	0.6	0, 1.2	1.8	0.9, 2.7	0	0, 0
6–8	1.2	0.4, 2.0	0	0, 0	1.8	1.0, 2.6	2.4	0.2, 4.6	11.2	8.0, 14.4	36.5	24.2, 48.8	8.0	3.5, 12.5	5.6	1.7, 9.5
9–11	2.2	0, 4.4	0	0, 0	1.5	0.2, 2.8	1.7	0.4, 3.0	4.7	2.2, 7.2	14.4	2.6, 26.2	10.3	7.1, 13.5	22.5	10.7, 34.3
12–14	0	0, 0	38.2	15.1, 61.3	4.9	2.6, 7.2	0	0, 0	0	0, 0	0	0, 0	4.7	2.5, 6.9	9.7	3.7, 15.7
15–17	0	0, 0	0	0, 0	0	0, 0	0	0, 0	2.3	0.5, 4.1	0	0, 0	2.3	0.9, 3.7	0	0, 0
Avg. age at diagnosis in years (*SE*)	4.16 (.06)		7.53 (.17)		5.65 (.04)		4.68 (.03)		7.05 (.04)		7.22 (.03)		9.45 (.04)		10.22 (.05)	
Mood disorders																
Birth–2	0.3	0, 0.6	0.4	0, 0.8	0	0, 0	0	0, 0	0	0, 0	0	0, 0	0	0, 0	0	0, 0
3–5	0.4	0, 0.8	0	0, 0	2.1	1.0, 3.2	0	0, 0	0.7	0, 1.4	0	0, 0	2.9	0.5, 5.3	0	0, 0
6–8	0	0, 0	0	0, 0	0.4	0, 0.8	0	0, 0	3.1	1.7, 4.5	6.0	0.1, 11.9	0.8	0.3, 1.3	2.6	0.3, 4.9
9–11	0	0, 0	0	0, 0	1.5	0.1, 2.9	0	0, 0	3.3	1.3, 5.3	0.7	0, 1.5	0.8	0.3, 1.3	8.9	2.7, 15.1
12–14	0	0, 0	0	0, 0	1.9	0.5, 3.3	0	0, 0	1	0, 2.0	3.6	0, 7.4	4.5	2.1, 6.9	15.9	5.6, 26.2
15–17	0	0, 0	0	0, 0	1.8	0, 3.6	0	0, 0	1.6	0, 3.5	7.9	0, 16.4	5.9	3.0, 8.8	0	0, 0
Avg. age at diagnosis in years (*SE*)	3.06 (.10)		–		7.55 (.11)		–		8.87 (.07)		9.41 (.15)		11.09 (.09)		11.71 (.08)	
Learning disorders																
Birth–2	35.6	28.4, 42.8	29.3	19.2, 39.4	1.8	0.9, 2.7	7.8	3.2, 12.4	0.4	0.1, 0.7	0	0, 0	0.1	0, 0.2	4.9	0, 9.8
3–5	10.6	5.8, 15.4	17.4	7.4, 27.4	37.1	33.1, 41.1	20.8	14.0, 27.6	9.7	5.0, 14.4	18.6	6.9. 30.3	12.4	8.3, 16.5	8.7	1.1, 16.3
6–8	7.9	3.7, 12.1	0	0, 0	2.1	1.2, 3.0	20.0	10.0, 30.0	37.9	31.9, 43.9	32.3	20.5, 44.1	11.3	6.4, 16.2	13.1	6.3, 19.9
9–11	0	0, 0	1.1	0, 2.2	2.7	1.3, 4.1	8.5	2.4, 14.6	0	0, 0	0	0, 0	21.1	16.3, 25.9	5.1	0.5, 9.7
12–14	0	0, 0	0	0, 0	0.8	0.2, 1.4	0.7	0, 1.4	1.8	0.3, 3.3	0	0, 0	7.6	4.1, 11.1	7.2	2.6, 11.8
15–17	0	0, 0	0	0, 0	0	0, 0	0	0, 0	0	0, 0	0	0, 0	3.0	0.4, 5.6	1.0	0, 2.1
Avg. age at diagnosis in years (*SE*)	2.39 (.02)		2.35 (.04)		4.86 (.02)		4.87 (.04)		6.66 (.02)		5.64 (.03)		8.54 (.03)		7.15 (.11)	
ADHD																
Birth–2	24.2	16.6, 31.8	10.7	3.3, 18.1	0	0, 0	6.2	1.6, 10.8	0	0, 0	0	0, 0	1.4	0, 3.9	0	0, 0
3–5	5.2	1.3, 9.1	15.8	5.8, 25.8	18.4	15.6, 21.2	13.4	7.6, 19.2	8.8	5.9, 11.7	5.8	0.2, 11.4	6.5	3.7, 9.3	0	0, 0
6–8	8.7	4.3, 13.1	1.9	0, 3.8	7.0	4.2, 9.8	4.0	0.5, 7.5	45.9	39.3, 52.5	55.3	42.3, 68.3	26.2	19.9, 32.5	8.5	3.6, 13.4
9–11	3.0	0.7, 5.3	0	0, 0	0.8	0.3, 1.3	0	0, 0	2.7	0.6, 4.8	0	0, 0	19.2	14.4, 24.0	13.7	4.9, 22.5
12–14	0	0, 0	0	0, 0	0.6	0, 1.2	0	0, 0	0.6	0, 1.2	0	0, 0	5	2.8, 7.2	35.1	16.8, 53.4
15–17	0	0, 0	0	0, 0	0	0, 0	0	0, 0	0	0, 0	0	0, 0	3.8	1.2, 6.4	0	0, 0
Avg. age at diagnosis in years (*SE*)	3.01 (.04)		3.09 (.05)		4.86 (.02)		3.42 (.05)		6.46 (.01)		6.24 (.02)		8.30 (.03)		11.69 (.06)	

CI, confidence interval; *SE*, standard error.

**Figure 2:**
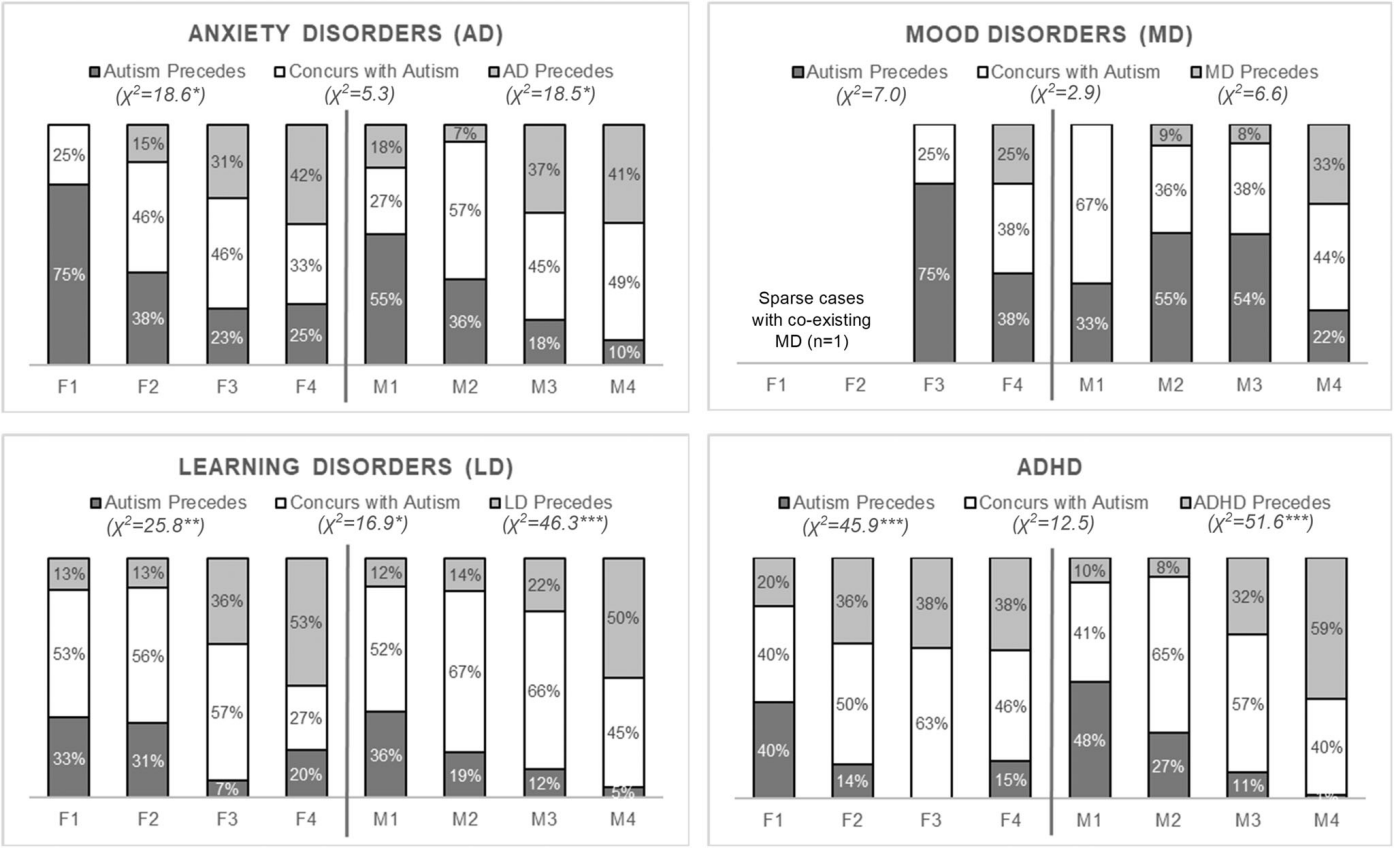
Proportions of participants with coexisting diagnoses after, concurrent with, or before autism diagnosis by latent class. The chi‐squared estimates presented below the legend labels indicate the overall group differences in the proportions for each category of temporal order for autism and the coexisting diagnosis (*p < .05, **p < .01, ***p < .001)

To further understand the timing of coexisting diagnoses in relation to autism diagnosis, we calculated the proportions of participants in each latent class with the coexisting diagnoses preceding, concurring with, or following the autism diagnosis. As shown in Figure 2, the proportions of coexisting diagnoses *concurring with* autism diagnosis were generally consistent across subgroups for all conditions [overall group difference: *χ* ^2^(7) = 2.9–16.9, all *p >* .01]. For anxiety disorders, LD, and ADHD, the proportions of autism diagnoses *preceding* coexisting diagnoses were higher in subgroups characterized by early autism diagnoses (Classes 1 and 2) compared to those with later autism diagnoses (Classes 3 and 4) [*χ* ^2^(7) = 18.5–51.6, all *p <* .01]. This trend was not evident for mood disorders, where the proportions did not differ significantly across subgroups [*χ* ^2^(6) = 6.6–7.0, *p >* .05]. Regarding sex differences, higher proportions with ADHD diagnosis *preceding* autism diagnosis (20%–36%) were observed in female classes characterized by autism diagnosed at preschool age (i.e., female Classes 1 and 2) compared to their male counterparts (8%–10%). The female subgroup characterized by autism diagnosed at age 9 and beyond (i.e., female Class 4) showed higher proportions of ADHD diagnoses *following* autism diagnoses (15%) compared to male Class 4 (1%). This is also reflected by the significantly higher average age of ADHD diagnosis in female Class 4 compared to male Class 4 (11.7 vs. 8.3 years, *p <* .001).

### Associated outcomes by latent class membership of initial diagnosis patterns

#### Multimorbidity status

Wald chi‐squared tests revealed significant overall differences in the number of coexisting conditions [*χ* ^2^(7) = 56.8, *p <* .001], coexisting MH‐NDD [*χ* ^2^(7) = 31.0, *p <* .001], MH‐only [*χ* ^2^(7) = 28.9, *p* < .001], and NDD‐only status [*χ* ^2^(7) = 17.6, *p* = .014] across male and female classes. A Poisson regression model, using male Class 2 as the reference group and controlling for age at sampling, showed that male and female Classes 3 and 4 had significantly more coexisting conditions (*z* = 2.1–3.1, all *p* < .05). Additionally, for coexisting MH‐NDD status, female Class 3 had significantly higher proportions of those with coexisting MH‐NDD compared to male Class 2 (50.8% vs. 13.0%; odds ratio [OR] = 6.8, *p* = .001). Weighted means and standard errors by latent class are reported in Table 4. Male Class 3 showed significantly higher proportions of children with NDD‐only compared to male Class 2 (58.6% vs. 40.4%; OR = 2.1, *p* = .021). No significant difference was observed for MH‐only status across classes.

**Table 4:** Multimorbidity status and functional difficulties by latent class (weighted average estimates with standard errors)

Associated outcomes		Multimorbidity status				Functional difficulties			
		Number of coexisting conditions	Coexisting MH‐NDD (%)	MH only (%)	NDD only (%)	Cognitive (%)	Behavioral‐Interpersonal (%)	Emotional (%)	Any (%)
Class 1	Male	1.04 (0.11)	10.35 (3.19)	1.04 (1.06)	48.71 (5.24)	58.29 (5.23)	62.92 (5.18)	24.85 (4.69)	76.25 (4.51)
	Female	1.01 (0.19)	12.09 (5.86)	9.85 (5.35)	42.22 (8.87)	55.94 (9.06)	55.52 (9.07)	24.81 (7.89)	77.48 (7.63)
Class 2	Male	0.90 (0.06)	12.96 (1.94)	3.37 (1.04)	40.43 (2.84)	41.56 (2.86)	59.02 (2.85)	26.16 (2.56)	70.18 (2.66)
	Female	1.10 (0.11)	22.18 (5.32)	9.26 (3.71)	40.47 (6.29)	57.69 (6.38)	68.89 (5.98)	24.48 (5.55)	73.11 (5.72)
Class 3	Male	1.35 (0.09)	15.15 (3.22)	6.55 (2.22)	58.59 (4.42)	35.54 (4.33)	66.79 (4.25)	31.85 (4.22)	79.13 (3.69)
	Female	1.73 (0.25)	50.76 (9.13)	0.85 (1.68)	19.30 (7.21)	49.12 (9.28)	68.06 (8.66)	47.35 (9.27)	80.98 (7.29)
Class 4	Male	1.55 (0.09)	27.39 (4.20)	5.32 (2.11)	51.35 (4.70)	40.79 (4.66)	75.06 (4.11)	37.83 (4.62)	80.90 (3.75)
	Female	1.60 (0.20)	22.19 (8.00)	19.87 (7.68)	45.28 (9.58)	51.14 (10.00)	96.10 (3.80)	51.79 (9.80)	98.15 (2.64)
Overall difference^a^ (*χ* ^2^, df = 7)		56.75***	31.00***	17.55*	28.86***	8.01	23.40**	14.84**	15.96**

MH, mental health conditions; NDD, neurodevelopmental conditions.

^a^ Wald chi‐squared tests for overall group difference, adjusted for age at sampling (*p < .05, **p < .01, ***p < .001).

#### Functional difficulties

Wald chi‐squared tests revealed significant overall group differences in behavioral‐interpersonal difficulties, emotional difficulties, and any difficulty [*χ* ^2^(7) = 14.8–23.4, all *p <* .01] across male and female classes, but not in cognitive difficulties. Using male Class 2 as the reference group and controlling for age at sampling, logistic regression analysis showed that male and female Classes 4 had significantly higher prevalence of behavioral‐interpersonal difficulties (ORs = 3.0 and 25.8, both *p* < .01), emotional difficulties (ORs = 2.7 and 5.2, both *p* < .05), and any difficulty (ORs = 2.6 and 35.1, both *p* < .01) compared to male Class 2. Weighted prevalence and standard errors by latent class are reported in Table 4.

## Discussion

In this study, we aimed to identify person‐centered subgroups based on the age at initial diagnoses for autism and four commonly coexisting mental health and neurodevelopmental conditions among autistic children and youth, from a nationally representative population‐based sample in Canada. In our sample, the prevalence of autism (2.0%) across the sample age range of 5–17 years was slightly lower than the estimate of 2.8% reported in the 2020 surveillance of 8‐year‐olds in the United States, while the female‐to‐male ratio (1:4.2) was comparable to the reported ratio of 1:3.8 (Maenner, 2023). Elevated prevalence of coexisting conditions was observed across conditions in the current sample. Notably, autistic females showed significantly higher prevalence of anxiety disorders (35.0%) compared to their male counterparts (18.8%). The current estimates for the mental health conditions were largely consistent with the previous meta‐analytic estimates for a similar age range (anxiety disorders ~20%, mood disorders ~5%, and ADHD ~30%; Lai et al., 2019; Mutluer et al., 2022). However, the current estimate for LD (48.7%) appears higher than prior population‐based estimates (23%) in autistic children and youth (Mutluer et al., 2022), yet lower than the estimates (60%–70%) reported in clinical samples of school‐aged autistic children (Estes, Rivera, Bryan, Cali, & Dawson, 2011; Mayes & Calhoun, 2006). This variation might also be due to differences in diagnostic indicators or criteria for LD across studies (Estes et al., 2011; Maki, Burns, & Sullivan, 2017). The average ages of initial diagnoses for neurodevelopmental conditions in our sample were 5–6 years, consistent with the peak age of initial diagnosis for any neurodevelopmental disorder (5.5 years) reported in recent meta‐analytic estimates across global general populations, but earlier than their estimates for ADHD (9.5 years) (Solmi et al., 2022). The average ages of mental health diagnoses in our sample were 6–9 years, which were much earlier than their peak estimates for any mental health disorder (14.5 years) in the general population (Solmi et al., 2022). Overall, our current population‐based estimates support the increased care needs for coexisting conditions among autistic children and youth.

To further understand the variability in the *timing* of diagnoses for autism and coexisting conditions, we adopted a multigroup approach for multivariate subgrouping. This resulted in the identification of four latent classes of initial diagnosis timing patterns, primarily distinguished by the age of the initial autism diagnosis. The largest class (Class 2) consisted of children and youth who were primarily diagnosed with autism at ages 3–5 years, aligned with the average or median age at autism diagnosis reported in prior population‐based studies (Maenner, 2023; Sheldrick, Maye, & Carter, 2017). The remaining three classes, characterized by an initial autism diagnosis primarily at birth to 2 years (Class 1), 6–8 years (Class 3), and 9 years and beyond (Class 4), each accounted for approximately 20% of our sample. However, a larger proportion of autistic females (20.8%) were classified into Class 1 compared to males (14.5%), while a greater proportion of males were classified into Class 2 (47.7%) than females (40.9%). No sex differences were observed in the prevalence of subgroups characterized by an elevated likelihood of autism diagnosis during school age (i.e., Classes 3 and 4). These observations suggest that among those diagnosed with autism at preschool age, females tended to receive an initial autism diagnosis *earlier* than boys. This is partially consistent with recent evidence from health records in the United States indicating that females tend to be diagnosed with autism either before age 3 or after age 11 years (Gu et al., 2023). This may be explained by a stronger association between the age of autism diagnosis and cognitive functional difficulties in females compared to males, suggesting that females in this very early diagnosis group may represent those with more profound autistic features who are often identified early in life, in contrast to their more cognitively able counterparts who may be diagnosed later (McDonnell et al., 2021). Our findings, which seemingly differ from previous reports of delayed diagnosis in autistic females (Harrop et al., 2024; McCormick et al., 2020), may highlight the advantage of the current subgrouping approach in revealing nuanced sex differences (especially in the distribution of age of diagnosis) that might be less apparent for specific age periods when using variable‐centered methods, such as comparing the average age of diagnosis between sexes across a broad age range. However, the small sample size of female participants in our study necessitates future replication to confirm these findings.

As shown in Figure 1, across these subgroups, elevated probability estimates for initial diagnoses of coexisting conditions generally aligned with the timing of autism diagnosis. Further examination of the diagnostic timing of autism and each coexisting condition revealed concurrence rates ranging from 37% to 59% across subgroups. An overall trend is that for subgroups diagnosed with autism early at preschool age (i.e., Classes 1 and 2), other conditions tended to be diagnosed concurrently with or after the autism diagnosis, whereas for subgroups with autism initially diagnosed in middle childhood through adolescence (i.e., Classes 3 and 4), other conditions were more often diagnosed concurrently with or before the autism diagnosis. However, the initial diagnosis of mood disorders tended to occur later in life, regardless of the age of autism diagnosis, which aligns with previous reports indicating that mood disorders are typically diagnosed during pre‐adolescence and adolescence in autistic people (Postorino, Vicari, & Mazzone, 2016).

Regarding sex differences in the timing patterns of coexisting diagnoses relative to autism, we found that a larger proportion (20%–36%) of autistic females diagnosed with autism at preschool age received an ADHD diagnosis *before* their autism diagnosis compared to their male counterparts (8%–10%). This observation is somewhat surprising, considering that attention or behavioral problems have typically been reported as more common and noticeable in males than in females (May, Cornish, & Rinehart, 2016). It is possible that when atypical behaviors are initially observed in females, they are more likely to be attributed to ADHD rather than to autism, given the previously reported high probability of shift from ADHD to autism diagnoses among girls with social and/or attention deficit (Kopp et al., 2023). A previous longitudinal study reported that females with autism diagnosed during early childhood showed more significant homotypic continuity in ADHD symptoms than males, indicating the early emergence of ADHD‐related signs in this subpopulation (Carter Leno et al., 2022). However, our results also revealed that females with autism diagnosed during adolescence tended to receive an ADHD diagnosis *later* than their male counterparts, with an average age of diagnosis at 11.7 years for females compared to 8.3 years for males. This delay might be related to camouflaging and/or diagnostic overshadowing of coexisting conditions often reported in this population (Lai, 2023; Lai et al., 2022). The previously documented greater delay in autism diagnosis for females with prior ADHD diagnoses compared to those without, relative to males (Kentrou et al., 2019), may be driven by those diagnosed with autism during adolescence or later in life. Further longitudinal research is warranted to elucidate the sex differences in the temporal order and reciprocal influence of autism and ADHD diagnoses.

Overall, subgroups with a later autism diagnosis beyond preschool age (i.e., age 6 and beyond) tended to have more coexisting conditions, especially mental health diagnoses, consistent with previous evidence (Jadav & Bal, 2022; Mandy et al., 2022; Smith et al., 2024; Soke, Maenner, Christensen, Kurzius‐Spencer, & Schieve, 2018). Notably, all female subgroups consistently showed higher probabilities of initial diagnoses of anxiety across ages compared to their male counterparts, aligning with prior findings from a large autism cohort study of over 5,000 autistic females (Wodka et al., 2022). Further, females with a later autism diagnosis during school age showed elevated probabilities of mood disorders diagnosed concurrently or afterward. Particularly, the female subgroup (i.e., female Class 3) diagnosed with autism at an early school age (6–8 years) had the highest average total number of coexisting conditions, with 51% having both coexisting mental health and neurodevelopmental conditions, and 47% reporting daily emotional difficulties. In contrast, their male counterparts (i.e., male Class 3) were more likely to have coexisting neurodevelopmental conditions alone, and the rate of emotional difficulties in this subgroup did not differ significantly from those diagnosed with autism during preschool age (i.e., male Classes 1 and 2). Prior research has reported higher prevalence estimates of coexisting neurodevelopmental conditions among those with childhood‐onset mental health conditions compared to those with adolescent‐onset ones (Doering et al., 2022), which may be explained by the stronger phenotypic correlations between neurodevelopmental and mental health conditions in childhood (Lundström et al., 2011). Another notable observation is that females diagnosed with autism by age 3 years showed an increased probability for an initial diagnosis of anxiety disorder at preschool age, as well as later in adolescence. This finding might be explained by prior longitudinal evidence indicating that children diagnosed with autism in toddlerhood with lower cognitive functioning are more likely to have separation anxiety, but not generalized anxiety, in adolescence (Ben‐Itzchak, Koller, & Zachor, 2020). Thus, the nature of anxiety may vary by diagnostic timing, warranting further research. Despite this potential variation, our findings suggest that autistic females may be at elevated risk for anxiety disorders regardless of the age at which autism is initially diagnosed. These findings highlight the importance of early detection and continuous monitoring of mental health needs for autistic people, especially females, irrespective of when they are diagnosed with autism, as well as providing support tailored to individual needs (Lai et al., 2023).

Furthermore, we examined subgroup differences in functional difficulties at the time of the survey. Results showed that children and youth diagnosed with autism in late childhood or adolescence were more likely to experience challenges in behavioral‐interpersonal and emotional domains. Notably, nearly all females in the subgroup characterized by late autism diagnoses (i.e., female Class 4; 96% compared to 56%–75% in the other subgroups) reported behavioral‐interpersonal difficulties, such as adapting to change and making friends, which are closely linked to core autism features. About half of the female subgroups diagnosed with autism at school age and beyond (i.e., female Classes 3 and 4) were reported to experience anxiety or depression symptoms on a daily basis. In contrast, although 75% of males in the late autism diagnosis group (i.e., male Class 4) had behavioral‐interpersonal difficulties, only 38% reported having emotional difficulties. This observation may be linked to previous findings suggesting more elevated autistic traits in females diagnosed later compared to their male counterparts. Females diagnosed later often recall that their childhood autism features become more impairing during adolescence (Bargiela, Steward, & Mandy, 2016; Davidovitch, Gazit, Patalon, Leitner, & Rotem, 2023; Mandy, Pellicano, St Pourcain, Skuse, & Heron, 2018). These findings emphasize the heightened mental health needs of later‐diagnosed autistic females, which may be associated with the widening person‐environment gaps during later stages of life, when social relationships and environmental adaptation become more challenging in less structured settings (Lai, Anagnostou, Wiznitzer, Allison, & Baron‐Cohen, 2020; Mandy et al., 2022).

The current study, applying a person‐centered approach that accounts for the timing of diagnoses, provides a complementary and nuanced perspective to the predominantly variable‐centered evidence in the literature. The mixture modeling approach is particularly well suited to uncover the intricate relationships between age at autism diagnoses, coexisting conditions, and sex assigned at birth. While our findings are consistent with some previous research indicating that autistic females are at elevated risk for internalizing mental health conditions such as anxiety (Hull et al., 2017), the association with age of autism diagnosis is not necessarily linear (i.e., older age of autism diagnosis is not always associated with a higher likelihood of mental health diagnosis) and is mostly unknown and under‐investigated. Specifically, females diagnosed very early (before age 3) or during school age showed elevated probabilities of receiving an anxiety diagnosis, either concurrently or later in life. We also observed that those with autism diagnosed during school age, particularly females, were more vulnerable to functional difficulties across emotional and behavioral‐interpersonal domains, with those diagnosed at early school age being most likely to have both mental health and neurodevelopmental diagnoses other than autism. These findings highlight the need for developing tailored interventions to address the mental health needs of autistic children and youth, focusing on: (1) providing integrated care or services for multiple types of conditions across mental health and neurodevelopmental conditions, (2) integrating services into primary care and school settings to facilitate timely identification of needs and early intervention efforts, (3) adopting a neurodivergence‐informed approach to designing and providing mental health care, and (4) addressing the unique needs of autistic females by considering variations in diagnostic timing and the functional impacts of coexisting conditions to provide tailored support (Kester & Lucyshyn, 2018; Lai, 2023; Lai et al., 2020, 2023; Pemovska et al., 2024).

There are several limitations to consider when interpreting the current findings. First, due to the cross‐sectional nature of the study, we cannot establish causality or directionality for the observed patterns. While we used information on the age at diagnosis to examine the temporal order of autism and coexisting health conditions, the lack of longitudinal data limits our ability to determine whether the onset of one condition influences the emergence and persistence of another or to account for potential changes in diagnostic status over time. The potential confounding effect of age at sampling cannot be entirely ruled out, despite controlling for it as a covariate in the analyses. Functional difficulties at the time of sampling were treated as dependent outcomes for subgroup comparisons, but it is important to consider intellectual functioning at the time of autism and coexisting diagnoses, which was not available in this dataset. This study also did not examine other mental health conditions with elevated prevalence in the autistic population, such as feeding and eating disorders or schizophrenia spectrum disorders (Lai et al., 2019; Micai et al., 2023), which may complicate the diagnostic process, particularly among those diagnosed with autism later in life. Further, we were constrained in validating the accuracy of caregiver‐reported diagnoses, as there may be recall bias, overreporting of undiagnosed concerns as actual diagnoses, or confusion between different diagnoses by respondents. Additionally, families with children who have severe disabilities may be less able to respond to the survey, potentially leading to sampling bias.

It should also be noted that the sample sizes for certain female subgroups were relatively small, potentially limiting the power to detect group differences and restricting our ability to unveil additional subgroups. Future replication studies with larger samples and greater representation of females are warranted. Given that the current sample reflects the general population and adheres to the ~4:1 male‐to‐female ratio of autism diagnoses (Loomes, Hull, & Mandy, 2017), increasing the female sample size would necessitate expanding the overall population‐based sample. Further, the current study used only sex assigned at birth as the grouping variable, without incorporating gender‐related information, due to the challenge of small sample sizes for subgroup analysis. Participatory research methods are also needed to incorporate the views of those with lived experiences in shaping terminology (e.g., alternatives to ‘multimorbidity’) and interpreting the implications of findings.

Despite these limitations, the current study represents a rare effort to map the initial diagnosis patterns of autism and commonly coexisting conditions using a person‐centered approach, offering insights that might not be fully captured by previous evidence derived from conventional variable‐centered methods. Importantly, the current sample was drawn from a representative Canadian population and therefore reflects real‐world health needs among autistic children and youth across the country. Future prospective studies tracking the timing of initial diagnoses and stability of these diagnoses over time, and their associations with demographic and socio‐economic characteristics, are needed for understanding the mechanisms influencing diagnostic timing to improve timely identification and support for the diverse neurodevelopmental and mental health needs of autistic children and youth.

## Conclusion

Our findings contribute to existing evidence primarily derived from clinical samples, showing elevated prevalence of coexisting mental health and neurodevelopmental conditions among autistic children and youth in the general population, with notably higher estimates for anxiety disorders observed in autistic females. While average ages of autism and coexisting diagnosis did not differ between males and females across our sample, nuanced variations emerged when examining subgroups based on the timing of receiving the initial diagnosis of autism and coexisting conditions identified through a person‐centered analytic approach. The age of autism diagnosis serves as a key indicator for subgroup distinction, with those diagnosed with autism during school age showing increased probability of mental health challenges. Notably, the sex‐varying patterns highlight the elevated mental health needs of autistic females, irrespective of the age of autism diagnosis. Females with autism diagnosed in middle childhood through adolescence are more likely to have multiple coexisting mental health and neurodevelopmental conditions, along with elevated behavioral‐interpersonal and emotional difficulties. These findings emphasize the importance of continuous monitoring, screening, timely detection, and early intervention for mental health and neurodevelopmental needs among autistic children and youth, particularly females, with support tailored to sex and age at the time of autism diagnosis.

## Ethical considerations

Participant confidentiality is protected under the Statistics Act, and data integrity is ensured by Statistics Canada. Informed consent, obtained by Statistics Canada, included permission for secondary data analysis, with all data in the analytic file deidentified. Thus, this study is exempt from ethics review by our institutional research ethics board in accordance with the Tri‐Council Policy Statement.

**Key points**

- Coexisting health conditions are common among autistic people, but little is known about how the timing of their initial diagnoses varies, particularly among those sampled from the general population.
- Using a person‐centered approach, we demonstrated that the age of autism diagnosis is strongly associated with the patterning of initial diagnoses of coexisting mental health and neurodevelopmental conditions, with differences observed between autistic males and females.
- Subpopulations with autism diagnosed in middle childhood through adolescence tend to receive multiple diagnoses, with females being more likely than males to have coexisting mental health diagnoses, as well as emotional and behavioral‐interpersonal difficulties.
- Timely detection and continuous monitoring of emotional and behavioral needs of autistic children and youth, with support tailored to sex and timing of autism diagnosis, are essential.

## Supporting information


**Table S1.** Standardized factor loadings from the confirmatory factor analysis model of selected WG/UNICEF CFM items in the current autistic sample.

## Data Availability

The data supporting the findings of this study are available from Statistics Canada through the Research Data Centre program (https://www.statcan.gc.ca/en/microdata/data‐centres/access), subject to approval.
