# Supplementary material for: Initial diagnosis patterns of coexisting mental health and neurodevelopmental conditions in autistic children and youth: Evidence from a nationally representative sample in Canada
Source: J Child Psychol Psychiatry. 2025 Sep 1;67(2):238–52. doi: 10.1111/jcpp.70039 (PMC12812786; doi:10.1111/jcpp.70039)
Supplement: Supplementary file 1 — Table S1. Standardized factor loadings from the confirmatory factor analysis model of selected WG/UNICEF CFM items in the current autistic sample. [file JCPP-67-238-s001.docx]

**Supporting Information**

**Table S1.** Standardized factor loadings from the confirmatory factor analysis model of selected WG/UNICEF CFM items in the current autistic sample

| **Factor**  **Items** | **Factor 1:** Cognitive | **Factor 2:**  Behavioural-Interpersonal | **Factor 3:** Emotional |
| --- | --- | --- | --- |
| Selfcare | .85 |  |  |
| Communication | .82 |  |  |
| Learning | .83 |  |  |
| Remembering | .69 |  |  |
| Concentrating | .68 |  |  |
| Accepting changes |  | .69 |  |
| Controlling behaviour |  | .81 |  |
| Making friends |  | .63 |  |
| Depression |  |  | .70 |
| Anxiety |  |  | .97 |
